# Perceptions, beliefs, and experiences of living with osteoporosis among UK South Asian older women

**DOI:** 10.1007/s11657-026-01726-5

**Published:** 2026-06-16

**Authors:** Shabana Shafiq, Nicky Kime, Jessica Faye Johansson, Elizabeth Teale, Janine Connor, Christine Sharp, Anne Heaven

**Affiliations:** 1 Academic Unit for Ageing and Stroke Research, Bradford Teaching Hospitals NHS Foundation Trust, Temple Bank House, Bradford, BD9 6RJ UK; 2 Bradford Institute for Health Research, Academic Unit for Ageing and Stroke Research, Bradford Teaching Hospitals Foundation Trust, Bradford, West Yorkshire UK; 3 Fragility Fracture Liaison Services, Orthopaedics Department, Bradford Royal Infirmary, Bradford, West Yorkshire UK; 4 Bradford Teaching Hospitals Foundation Trust, Bradford, UK

**Keywords:** South Asian, Bone health, Qualitative, Health beliefs, Primary care services, Older Women

## Abstract

***Summary*:**

Older women of a South Asian heritage are at higher risk of osteoporosis yet remain less likely to access healthcare services for support with osteoporosis. Our study found sociocultural and religious factors shape their decisions. Designing culturally tailored services can help South Asian older women access timely osteoporosis support.

**Purpose:**

To explore South Asian older women’s beliefs and experiences of living with osteoporosis, with the aim of informing health service delivery.

**Methods:**

Eleven older women with a self-reported diagnosis of osteoporosis were conveniently selected from community organizations in West Yorkshire, UK. Data were collected between September 2024 and March 2025. In-depth semi-structured interviews were audio recorded, transcribed, and analyzed using constructivist grounded theory.

**Results:**

Four themes were identified; a perceived natural physical deterioration, beliefs about causes of osteoporosis, impact of osteoporosis, and adapting to living with osteoporosis. Osteoporosis was perceived as natural physical deterioration which occurred as an inevitable part of the ageing process. It was also perceived to be associated with existing health conditions and viewed as ordained by God. Osteoporosis impacted lives of South Asian older women by causing loss of familiar routine, loneliness and social isolation, and negative emotions. However, South Asian older women adapted to living with osteoporosis through family-based support, traditional remedies and practices, and using religion as a coping strategy.

**Conclusion:**

South Asian older women’s sociocultural perspectives and emotional and coping strategies for managing osteoporosis influenced decisions to access healthcare services. It is imperative for health providers to consider the individual meaning of osteoporosis for this population in relation to their specific needs, to improve engagement with osteoporosis healthcare and support services.

## Introduction

Osteoporosis is characterized by reduced bone mineral density which results in bone fragility and increased risk of fracture [1]. In England and Wales, more than two million women have osteoporosis [2]. Osteoporosis leads to around 180,000 fractures annually causing significant pain and disability, affecting women’s independence and quality of life [2]. If osteoporosis is not treated and effectively managed, over one in 20 patients is likely to suffer a second fracture within four years [3]. This is crucial given the increasing effectiveness of fracture prevention with advancing age [4].

Current guidelines by the UK National Osteoporosis Guideline Group (NOGG) and Royal Osteoporosis Society (ROS) recommend timely diagnosis, treatment and management of osteoporosis in primary care, for the prevention and reduction of secondary fragility fractures [5, 6]. This includes appropriate pain assessment and pharmacological treatment, provision of nutritional advice and supplements, support with implementation of weight-bearing exercise and lifestyle changes, and timely referral to appropriate physical and emotional support services to reduce falls risk and improve psychological wellbeing [7–9].

South Asian (SA) people of Pakistani, Indian, and Bangladeshi heritage are the largest minority ethnic population in the UK [10]. Women aged 65 + account for approximately 4% of this population [11]. Older SA women are at an increased risk of osteoporosis compared to their White British counterparts due to several risk factors which impact bone health, leading to poorer health outcomes in later life for this population group [12]. These include higher vitamin D deficiency, earlier bone resorption, poor diet, lower physical activity, and greater prevalence of co-morbidities including diabetes and cardiovascular disease [13, 14]. However, despite their higher risk factors, SA older women remain less likely to access healthcare services in general, including those for managing osteoporosis [15, 16]. This is problematic and can result in their support needs being unmet, thus impeding their overall health, wellbeing, and quality of life [17].

While previous research by Mckenna and Ludwig [13] investigated UK SA women’s care experiences when living with osteoporosis, subsequent literature has focused on assessing bone resorption levels in child-bearing aged and pre/post-menopausal SA women [18–21]. Little is known about the specific perceptions and experiences of UK first-generation SA older women and how these influence decisions to seek osteoporosis support from primary and secondary healthcare services. The SA population makes up the largest ethnic minority group of the West Yorkshire region [11] and 18% of older women with fractures belong to a minority ethnic group [22]. Our study was part of a wider NIHR-funded national study which aimed to use insights from the experiences of older women and primary healthcare professionals to develop strategies to improve osteoporosis care. The aim of our sub-group analysis was therefore to investigate SA older women’s perceptions, beliefs and experiences of living with osteoporosis. This could help inform health services and improve timely access to support for managing bone health and wellbeing in later life for SA older women living with osteoporosis.

## Methods

### Design

We employed a qualitative design.

### Study setting and recruitment

Our sub-study was conducted across the West Yorkshire region of the UK. The first author (SS) visited and provided flyers and an information sheet containing study details to representatives of SA community organizations in West Yorkshire. We used a convenience sampling strategy to identify and select older women aged 70 + with a self-reported diagnosis of osteoporosis and living in the community. We did not include care home residents or women with a diagnosis of dementia as they would require different interventions to community-dwelling older women and those without dementia. SS received referrals of interested participants from community organization representatives and contacted them by telephone and face to face. The information sheet was read and explained to participants in community languages to ensure informed consent and sufficient knowledge and understanding of the study. Participants were also advised of the cooling-off period should they have wished to withdraw from the study without any consequence. Interested participants were next screened for eligibility based on the study inclusion/exclusion criterion (Table 1) and written informed consent acquired from all who agreed to participate.

**Table 1:** Participant inclusion/exclusion criterion

Inclusion	Exclusion
Any SA ethnic background	Non-SA ethnicity
Aged 70 years or older	Aged under 70 years
Self-reported osteoporosis diagnosis	No osteoporosis diagnosis
Residing within the community	Resident in a care home
Capacity to consent to participate in research	Lacks capacity to consent to participate in research
	Diagnosis of dementia

### Data collection

Semi-structured individual and dyad interviews were conducted in English, Urdu, Punjabi, and Hindi between September 2024 and March 2025. Interviews took place in participant’s homes, community settings, and via telephone, according to SA older women’s preferences. All interviews lasted up to 90 min. The focus of the interviews broadly included domains relating to National Osteoporosis Guideline Group and Royal Osteoporosis Society recommendations and iteratively shaped by discussions with our co-production group comprising SA older women with osteoporosis. All interviews were audio recorded using an encrypted voice recorder, and field notes made. Participants received a £10 voucher for their time.

### Data analysis

Interviews in English were recorded and transcribed by a transcription service. Those conducted in Urdu, Punjabi, and Hindi were translated into English and transcribed by SS. Those conducted in Urdu, Punjabi, and Hindi were translated into English and transcribed verbatim by SS, with careful consideration for nuances in language. Words which carried different meanings in each language were translated to the closest equivalent (e.g., “alag thalag” translated to “isolated,” “buzurg/bujurg log” translated to “elderly” and “baagbaan” translated to “watering plants”) to ensure significance and context were not lost. All interview recordings were relistened to by SS to check for accuracy and enable data immersion. A five-step constructivist grounded theory analysis process was then used to create inductive themes [23]. Initial line-by-line coding was undertaken by SS after which direct, selective, and conceptual codes were iteratively formed with AH and NK through discussion. Next, field notes and initial and focused codes were reflected upon by the research team and members of the co-production group to compare data. Focused codes and memos were then collapsed into categories for synthesis after which SS, AH, and NK collaboratively developed inductive themes and sub-themes. Microsoft Word, Excel, and NVivo software were used to manage, facilitate, and record data analysis.

### Rigor

To increase rigor and trustworthiness of findings, we engaged in regular team reflexivity to reduce bias and assumptions and reviewed categories and themes with the co-production group. Encompassing objectivity from all involved was particularly valuable for reducing any potential bias stemming from SS’s position as an insider researcher, thus contributing to much improved accurateness of the research findings.

### Ethical consideration

We obtained ethical approval for the wider study by the London Bromley Research Ethics Committee on 21st May 2024 (24/PR/0500). Participants were assured of anonymity and confidentiality, and data securely stored on a password-protected device.

## Results

A total of 11 SA older women participated in individual (*n* = 5) and dyad interviews (*n* = 3). Ages ranged between 70 and 87. Participants were of Pakistani Muslim, Indian Sikh, and Indian Hindu background (see Table 2).

**Table 2:** SA older women demographics

Participant ID	Age	Years since diagnosis	Ethnic background	Religion	Language spoken
OW014	74	Unknown	Pakistan	Muslim	Punjabi
OW015	73	4	Pakistani	Muslim	Punjabi & Urdu
OW016	70	14	Indian	Sikh	English
OW018	73	10 +	Indian	Sikh	Punjabi
OW020	83	2	Indian	Hindu	Hindi
OW023	70	7	Indian	Hindu	Hindi
OW024	73	24	Indian	Hindu	Hindi
OW025	86	8	Indian	Hindu	Hindi
OW026	87	2	Indian	Hindu	English
OW028	80	7	Indian	Hindu	Hindi
OW029	70	6	Indian	Hindu	English

Four themes were identified from the data. These included a perceived natural physical deterioration, beliefs about causes of osteoporosis, impact of osteoporosis, and adapting to living with osteoporosis. Direct quotes have been used to provide context.

### A perceived natural physical deterioration

Many SA older women conceptualized osteoporosis as a natural, physical deterioration which was expected to occur as part of the ageing process. Participants attributed the physical symptoms they experienced to growing older, as opposed to symptoms of osteoporosis. For them, osteoporosis symptoms of bone loss and reduced height were a natural part of growing older, which could not be prevented or managed.

I struggle with my bones. But these things happen when you get old don’t they?” (014).

It’s a bone condition, isn’t it? So, the older you get, I suppose it’s going to affect you more (027).

I always thought that I was just getting shorter… just thought it was related to age (020).

For some SA older women, physical symptoms of osteoporosis were perceived as reduced muscle capacity in older age. These perceptions were often shaped by conversations with family members which they felt informed their perceived knowledge and understanding of the physical symptoms of osteoporosis.

My son told me it’s weak muscles… deterioration of the muscles (025).

### Beliefs about causes of osteoporosis

SA older women expressed varied perceptions and beliefs about the causes of osteoporosis. They linked the condition to past fertility experiences, long-term health conditions and disease, lifestyle, COVID-19 vaccinations, and ordained by God.

### Past gynaecological health experiences

Some women attributed their bone deterioration to previous hysterectomies following childbirth complications during their younger years, which led to osteoporosis. One woman explained this as,

When I was younger, they had to do a hysterectomy on me after the second caesarean. After the hysterectomy I began to suffer so much with my bones, my body deteriorated a lot…obviously the bones and everything in your body get weaker you know” (014)

### Existing long-term health conditions and disease

Many SA older women associated osteoporosis with receiving treatment for existing long-term conditions, diagnosis of disease, and neurological symptoms. They felt that these experiences had had a profound impact on their bone health and caused osteoporosis,

I’ve been diabetic for 40 years now … using insulin injections for 20 years for my diabetes … When you’ve been diabetic for a long time it’s more likely that your bones will get weaker and can break (018)

I think it was the cancer that had caused it (015)

Before my one hand used to shake and now … my head has started to shake so it makes me think that maybe my bones have become damaged because of this condition (018)

### COVID-19 vaccine

Some older women attributed osteoporosis to receiving COVID-19 vaccinations, believing these had worsened their bone health by affecting their physical strength and generating feelings of weakness. One participant expressed this as,

I think the two covid jabs I had made my bones worse. It has made me feel that my bones no longer have any strength in them … always lose my strength after it and feel weak in my body (014)

### Lifestyle

Some SA older women perceived causal factors of osteoporosis as related to lifestyle, which could be controlled through healthy nutrition and reduced alcohol consumption, though the latter was specifically related to older women of Indian heritage. Here, participants felt their infrequent consumption of alcohol did not contribute toward their osteoporosis,

Just small amounts of alcohol, very little (023)

Sometimes I drink wine… only when in party. I’m not drinker nah (028)

### God ordained

For many SA older women, spiritual beliefs shaped their perceptions of causality of osteoporosis. Several participants perceived osteoporosis as God-ordained and viewed the condition as part of divine will and a greater plan, which was unable to be prevented or controlled.

You see, everything is from God Almighty, I am nothing. It’s all from only God (014)

I’ve been destined this from Allah (God) so for everything Allah is the planner for me … [for] the health condition bestowed upon me (015)

Consequently, many women chose to prioritize other health conditions such as diabetes or prolapse over bone health with little concern for their osteoporosis,

I’m more worried about my other conditions… I’m suffering more with that (016).

For me, my diabetes is more important (018).

### Impact of osteoporosis

SA older women reported many negative impacts of osteoporosis. This included loss of familiar routine, experiences of negative emotions, and loneliness and social isolation.

### Loss of familiar routine

Many older women experienced a loss of ability to conduct activities of daily living chores as an impact of osteoporosis. For them, the unpredictability of symptoms and fluctuating nature of the condition led to a decline in their ability to carry out activities of daily living household activities, which were once solely achievable. Participants expressed this as,

I’m not as active as I used to be for my daily tasks for the housework, you know, one day might be my back is quite bad, another day my necks are bad, another day my hands are bad (016)

I can’t do any household chores … can’t even make chapatti for myself

### Negative emotions

Most SA older women reported experiencing negative emotions as an impact of osteoporosis. They expressed a constant fear of falls and fractures and potential for sustaining further injuries because of their poor bone health. This was stated as,

It’s frightening because it’s my bones that are weakened … you know, if I do fall, I don’t want to fracture anything (016)

I feel so scared that I’ll fall and hurt myself or break my bones. I’m worried because my bones have become very weak so that’s what causes my fear [of falling] (023)

Another participant expressed how experiencing a loss of trust in their own physical resilience generated higher levels of anxiety, leading to increased reliance on others for support.

I feel scared to go out on my own in case I fall and break another bone. I’m very worried about going out alone because of that. I can only go out if I have someone with me (024)

### Loneliness and social isolation

Some SA older women experienced loneliness as an impact of osteoporosis, particularly when they were unable to rely on family members for support in managing daily tasks. One participant described how the physical absence and work commitments of family members across geographically dispersed locations generated feelings of loneliness when struggling to cope with their everyday challenges related to osteoporosis,

I have my son only to help, no one else, but he lives far away so I’m all alone. It’s difficult, difficult. My family is really small you see, and they all live in Leicester, I have no-one else here. So just my son helps me, usually by telephone. It’s a very small family I come from, small family. My son doesn’t have much time because he’s working, but he telephones me every day to check in on me (025)

Many SA older women also described experiencing feelings of isolation and disruption to their social routines due to osteoporosis limiting their mobility and causing difficulties in participating in previously enjoyable activities,

I used to enjoy gardening, used to do that quite a lot, and now I cannot even do that. Planting, watering plants, I used to enjoy do it all. But recently I’ve not manged to look after my plants, haven’t been able to water them so now they’ve died, poor things (018)

Others reported how osteoporosis not only affected their ability to maintain regular social interactions but also contributed to feelings of loneliness and social isolation through absence of support networks and disconnection from their communities,

Feel isolated and more lonely, if they haven’t got any...support from anybody or like that, it is very very lonely, that you are managing on your own but you haven’t got a back-up with it (016).

I wish I could do what I used to do before… I don’t like to stay indoors for too long (018).

### Adapting to living with osteoporosis

SA older women reported adopting many strategies to help cope with and adapt to living with osteoporosis. These included drawing on family support, using religion as a coping mechanism, and utilizing traditional remedies and practices to self-manage their bone health.

### Family-based support

Most SA older women expressed how receiving support with daily chores from family members within multi-generation households enabled them to adapt to living with osteoporosis. They reported how familial support allowed them to self-manage both the condition and activities of daily living with greater ease and comfort,

I tell my granddaughter for help with the chores [laughs]. I have my daughter and granddaughter to support me. My granddaughter lives with me, she is a big help (023)

I have my husband’s support so that’s enough for me. Whether he wishes to cook or bring a cooked meal from outside! [laughs] … From the beginning we’ve had a very good life together... [smiles] (018)

Others perceived support provision from adult family members including daughters and daughters-in-law as key to managing daily life, medication management, and communication with healthcare professionals in relation to their osteoporosis,

My daughter in-law, any household chores that I need support with she helps me. She does the cooking, cleaning, everything, because I cannot do anything at all. She helps me take my medication too. My daughter manages it on my behalf and daughter in-law helps administer it. My daughter manages all my medication needs by telephone with the doctor. She will ring the doctor and speak to him about it (024)

### Religious coping strategies

Many SA older women reported using religion as a source of emotional strength and acceptance, which helped them to make sense of deteriorating health in older age and accept osteoporosis as part of God’s blessings. Here, religious belief served as a powerful coping mechanism through beliefs that ill health was part of a larger meaningful journey in which older women were unable to personally manage their health and would receive support from God alone,

You know I am an old woman now, so I rely on Allah to help me through life with all my conditions, He’s the best to support me… You see, everything is a blessing from God Almighty, I am nothing so only God can help me in times of ill health (014)

Some Muslim older women described adapting to living with osteoporosis through beliefs that ill health was part of divine destiny, choosing to respond to the condition with positivity and joy,

Alhamdulillah, Alhamdulillah [gestures prayer sign with hands] I’ve been destined this from Allah so instead of crying about it why not just accept it and live life full of laughter and happiness? (015)

One Indian Sikh older woman expressed gratitude, believing that continuing to serve in the Gurdwara led to spiritual comfort and God’s divine mercy. For her, reframing osteoporosis as preparation for an eventual return to God enabled acceptance and adjustment to physical deterioration and demanding chores when adapting to familiar faith based activities,

I thank God for everything I have. My husband told me that I’ve not to continue working at the kitchen [in the Gurdwara] again, don’t carry out any hard work activities, washing dishes and stuff. I thought to myself that I am serving God that I may receive His mercies … don’t know when I’ll be called back to Him. So, I’ll make some meals for the Langar but at a really really slow pace (018)

Other older women shared how religion helped adapt to living with osteoporosis through the belief that religion increased physical resilience via receiving direct support from God to prevent falls and fractures,

ShukrAllhamdulillah [Glory be to God] I can’t praise God enough; He saved me from having broken bones and fractures despite falling so many times (014)

### Traditional remedies and practices

For some SA older women, cultural background and country of origin informed their attitude toward self-management of fractures when adapting to living with osteoporosis. One woman referred to her cultural norm of managing health within the household and belief that medical consultation was unnecessary for bone health.

I just massaged my ankle, wrapped bandages around the fracture. I had got used to wrapping my foot with bandages in Pakistan. Whoever went to the doctors back in those days? Everyone just managed at home (014)

Others described their confidence in the perceived effectiveness of using traditional herbal remedies to self-manage bone health, due to their cultural belief that turmeric was central to improving bone health because of its anti-inflammatory and healing properties,

Sometimes I use, you know, Haldi, like turmeric and that helps a lot (029)

Many SA older women further reported using varied methods to seek information on treatment and nutritional advice for improving bone health through culturally accessible social media channels and written materials. For them, culturally tailored written materials created by SA health experts were considered valuable for adapting to living with osteoporosis as they felt able to resonate with and trust in such culturally relevant sources,

I look on the internet …view videos about juices to drink to strengthen your bones, which diet is good, what to eat and what to avoid. They tell you about what’s good for people with brittle bones (018)

I keep reading about it in our Asian Voice, Gujarat Samachar and Asian Voice [magazine]. They write down about elderly people … what they have to do, how to manage (028)

## Discussion

To our knowledge, our study is the first in over a decade [13] to explore the beliefs and experiences of SA older women living with osteoporosis in the UK. SA older women’s sociocultural perspectives and access to health services were influenced by their beliefs and experiences, and emotional and coping strategies for living with and managing osteoporosis. Our findings demonstrate SA older women view osteoporosis as a natural physical decline which occurs as an inevitable apart of the ageing process. It is also perceived to be associated with previous gynaecological health experiences, existing health conditions and disease, vaccinations, lifestyle, and viewed as God ordained. Osteoporosis profoundly impacts the lives of SA older women by causing loss of familiar routine, isolation and loneliness, and negative emotions. However, SA older women believe it is possible to adapt to living with osteoporosis through drawing on family-based support, traditional practices and remedies, and using religion as a coping strategy.

Our findings align with broader literature indicating that older women from ethnic minority communities frequently interpret health symptoms as age-related [24–26], which can obscure recognition of symptoms of osteoporosis and lead to acceptance of the condition. Prior research has identified poor health literacy across ethnic minority groups [27, 28] which may hinder understanding of bone health and delay appropriate healthcare engagement for support with management of osteoporosis for SA older women. It is therefore important to develop culturally tailored health education and materials aimed at addressing misconceptions and improving awareness of osteoporosis as treatable and manageable at any age within this population. This may have the potential to not only improve early recognition of symptoms of osteoporosis but also promote timely access to healthcare services to support bone health management and wellbeing outcomes for SA older women living with osteoporosis.

SA older women in our study associated osteoporosis with gynaecological health history, chronic illnesses and lifestyle practices. This suggests good knowledge around risk factors of osteoporosis among this population group. However, notably, our study also identified beliefs linking osteoporosis to Covid-19 vaccinations, an association not previously documented in the literature. This finding echoes emerging evidence which demonstrates historical experiences of marginalization and distrust among minority ethnic groups can generate suspicion and skepticism toward vaccinations and related health conditions, with mistrust being profoundly heightened for older adults living with health conditions from SA communities [29–31]. This suggests that while SA older women have good knowledge of osteoporosis risk factors, this knowledge does not always translate into increased healthcare engagement for support with management of the condition due to structural inequalities, systemic barriers and limited culturally appropriate services which continue to hinder access for these communities [32, 33]. There is therefore a need for health services to adopt culturally responsive approaches which engage with the specific beliefs and concerns of SA older women living with osteoporosis. Addressing myths and misconceptions about osteoporosis and vaccinations through targeted health and community-led education initiatives may help build trust within this population. It is possible that this, in turn, may promote timely healthcare engagement and support effective management of bone health for SA older women living with osteoporosis.

Our findings further identified that spiritual beliefs played a significant role in shaping SA older women’s understanding of osteoporosis. This aligns with broader literature which show that SA individuals interpret illness through religious and spiritual frameworks, often viewing health conditions as divinely ordained [25, 28, 34, 35]. Like wider ethnic minority groups, religion is regarded as a preferred form of treatment control for long-term health conditions among SA older women which allows them to not only manage their emotional wellbeing but also offer a source of comfort during times of distress [36]. The perception of osteoporosis as uncontrollable or fated may thus discourage engagement with health services particularly if the condition is seen as beyond personal control [25]. To counteract this, culturally sensitive education that acknowledges spiritual beliefs while promoting manageability of osteoporosis is key. By addressing fatalistic attitudes through faith-informed health messaging SA older women may be more inclined to seek support and adopt preventive behaviours. This approach may not only improve osteoporosis outcomes for SA older women but also enable appropriate management of other long-term conditions which are also prevalent in this population.

Building on previous studies [25, 37, 38], our findings highlight the physical, social and emotional consequences of living with osteoporosis for SA older women. Osteoporosis significantly disrupted the wellbeing of SA older women by impacting their roles, routines, and relationships in profoundly personal and culturally meaningful ways. Wider evidence indicates that for older women across SA communities preparing food is considered an expression of care, tradition, and personal agency [39]. It is likely that experiencing a transition from caregiver to care recipient due to role reversal and dependency on others generated feelings of diminished self-worth and perceived erosion of identity among SA older women [40].

Findings reveal osteoporosis also imposed significant restrictions on social engagement for SA older women. This corroborates evidence which shows that within collectivist cultures time spent outside the home is closely linked to cultural participation and emotional wellbeing for SA older adults [37]. However, for SA older women, the condition may have limited access to spaces where they felt safe, active, and socially connected thereby exacerbating feelings of social exclusion and disconnection. Moreover, SA older women frequently felt distressed at their functional limitations and navigated their environments through fear, anxiety, and uncertainty. This corresponds with literature which indicates that SA women may experience heightened emotional distress when living with chronic health conditions, with good physical health often associated with improved emotional wellbeing in later life for this minority ethnic group [38]. It is therefore important for health services to recognise and understand the broader psychosocial impact of living with osteoporosis for SA older women. There is a need for health professionals to offer culturally sensitive support which acknowledges the intersection of health, identity, and cultural practices to appropriately support management of bone health in SA older women. It is possible that by tailoring services to facilitate autonomy through culturally appropriate physical, practical, and emotional support, SA women may deem them to be more appropriate and relevant. This may encourage greater engagement with services to improve health.

Our study found that like previous research [15, 25, 37, 41], SA older women adapted to living with osteoporosis through drawing on family-based support, cultural and traditional remedies and practices, and using faith as a coping strategy. Evidence suggests residing in multi-generational living and utilizing family-centred support plays a positive role in adapting to living with health conditions for SA older people. Older adults are culturally valued and respected within SA communities and receiving physical, emotional, and practical support from older adult children and extended family members has been shown to contribute to improved wellbeing and continuity of identity for SA older people [37, 42, 43]. However, while the familial structure makes available physical support and emotional reassurance, family members may also be a potential source of misinformation leading SA older women to not perceive a need to access health services for formal osteoporosis management.

Broader studies also show that beliefs around the need for and efficacy of traditional herbal remedies and cultural practices for health condition management are important for SA communities [44]. Use of herbal remedies and practices among this population group is commonly linked to older peoples’ desire to retain traditional practices, manage their acculturation struggles, and need to sustain connections with their countries of origin when living with long-term health conditions [44]. However, it can be argued that traditional herbal remedies may generate a sense of self-sufficiency among SA older women, reducing a perceived need for support from formal health services for appropriate management of osteoporosis. Additionally, studies demonstrate SA people hold preference for health and treatment information through modalities which recognize and are representative of their ethnicity and culture, due to feeling able to resonate with and trust in such culturally relevant resources [45]. While it is acknowledged that SA communities can gain health knowledge through culturally relevant materials [45] there is potential for risk of misinformation and inaccuracies across such channels. This can lead to poor effective management of osteoporosis and adverse health outcomes for SA women living with the condition.

Taken together, such factors may contribute to not accessing healthcare services for management of osteoporosis in SA older women. It is therefore important for health service providers to gain a deeper understanding of the specific coping strategies used by this population for managing and living with osteoporosis. There is a need to offer holistic support for SA older women which is inclusive of their cultural, traditional and social roles and practices. It is possible by doing so, interventions can be created incorporating these strategies to design culturally appropriate health care and support services which facilitate autonomy and wellbeing for SA older women living with osteoporosis in the UK.

## Strengths and limitations

Some limitations to our study are acknowledged. Our sample size was small and did not include participants from Bangladeshi backgrounds, thus limiting the generalizability of our findings across the broader UK SA community. Also, recruitment was confined to community services within a single region of the UK which can potentially exclude the beliefs and experiences of SA older women who do not engage with such services, and limit generalizability outside these contexts. Furthermore, although there was a concerted effort to increase credibility and reduce risk of losing meaning during the process of translating three different South Asian languages, some meaning may have become lost during the translation process.

Nevertheless, several strengths of our study are highlighted. Our study builds on limited literature exploring beliefs and experiences of first-generation UK SA older women with osteoporosis, a subgroup underrepresented in health research. Our participant cohort ranged in age from 70 to 83 and included women with diverse cultural and religious backgrounds. Our study design was informed by culturally sensitive methods including community-based recruitment and a multilingual researcher familiar with SA customs and practices. By acknowledging the intersection of age, ethnicity, gender and health status, our study offers a holistic view of SA older women’s beliefs and experiences to develop culturally tailored interventions and promote equitable healthcare support and services for SA older women living with osteoporosis in the UK.

## Conclusion

Our study identifies understanding beliefs and experiences and how these influence perceptions of osteoporosis in UK SA older women is important to build and shape healthcare support services. Health professionals and service providers need to consider the individual meaning of osteoporosis for this population in relation to their social, cultural, and religious context and beliefs. This can aid understanding of their specific support needs, which can then be integrated when planning and delivering culturally tailored osteoporosis care and support. There is also a need for development of interventions which focus on improving education in SA older women to reduce misconceptions and misinformation around osteoporosis. This may help improve timely access to primary and secondary care health services for specialist support in managing their physical, social and emotional wellbeing when living with the condition. Future studies can explore how SA older women with a diagnosis of osteoporosis navigate healthcare systems and how culturally sensitive interventions can be evaluated for effectiveness in improving health outcomes for this population group.
